# Community health assessment using self-organizing maps and geographic information systems

**DOI:** 10.1186/1476-072X-7-67

**Published:** 2008-12-30

**Authors:** Heather G Basara, May Yuan

**Affiliations:** 1 Center for Applied Social Research, University of Oklahoma, 101 David L. Boren Blvd., Norman, OK, USA; 2 Center for Spatial Analysis, University of Oklahoma, 300 Monitor Ave, Norman, OK, USA

## Abstract

**Background:**

From a public health perspective, a healthier community environment correlates with fewer occurrences of chronic or infectious diseases. Our premise is that community health is a non-linear function of environmental and socioeconomic effects that are not normally distributed among communities. The objective was to integrate multivariate data sets representing social, economic, and physical environmental factors to evaluate the hypothesis that communities with similar environmental characteristics exhibit similar distributions of disease.

**Results:**

The SOM algorithm used the intrinsic distributions of 92 environmental variables to classify 511 communities into five clusters. SOM determined clusters were reprojected to geographic space and compared with the distributions of several health outcomes. ANOVA results indicated that the variability between community clusters was significant with respect to the spatial distribution of disease occurrence.

**Conclusion:**

Our study demonstrated a positive relationship between environmental conditions and health outcomes in communities using the SOM-GIS method to overcome data and methodological challenges traditionally encountered in public health research. Results demonstrated that community health can be classified using environmental variables and that the SOM-GIS method may be applied to multivariate environmental health studies.

## Background

Population health can be viewed as a complex and dynamical system in which the patterns of health and disease exist, persist, and change over geography and time. [1,2] The underlying patterns of exposure that influence health status are the non-random result of interactions between the social, economic, and environmental networks people live within. [3] Therefore, understanding the macro-level effects of environmental determinants of health has become increasingly important. [4,5]

Epidemiologic studies have indicated that people and communities cluster spatially in systematic ways that are highly predictive of disease. [6] Such patterned regularity between groups and communities over time, despite the movement of people in and out of groups, demonstrates a dynamic at the environmental level that accounts for the observed differences in disease rates across spatial and temporal dimensions. [7,8] Epidemiologists have studied this dynamic interaction using complexity theory [9,2], in which populations are considered more than simply a collection of individuals but rather an important context that is fundamental for understanding the causative relationship between determinant and health outcomes. [3] Populations function within a highly composite, complex, adaptive system built up from large numbers of mutually interacting subunits whose repeated interactions result in rich, collective behaviour that feeds back into the behaviour of the individual parts. [2,10] Nonlinearity is the essence of complex systems. [11] Thus, challenges for conducting studies rooted in complexity arise when standard statistical modelling methods are applied to nonlinear and skewed data sets with interactive variables, hierarchical levels of analysis, and feedback mechanisms. The challenge is to understand the environment as it influences health outcomes by using analytical systems that are neither to simplified nor too complex. [12]

### Methods for Studying Complex Systems: The Self-Organizing Map

The self-organizing map algorithm (SOM) has been applied in medical research to address the need for non-linear analytical methods to study the multifaceted aetiology of certain diseases. Kohonen developed the algorithm to search for patterns within expansive, multivariate, numerical datasets. [13] SOM fit into the neural network class of methodologies and are tolerant of non-normally distributed data. Multivariate data sets can be developed to represent entities of interest for pattern recognition. Most recently, Oyana et al. applied SOM in a geospatial context to study cases of adult asthma. [14] Valkonen et al. used the SOM to explore the multidimensionality of insulin resistance syndrome. [15] In addition, neural networks have been applied to diagnose myocardial infarction, find patterns in genes, and organize genes according to biological relevance. [16-19] Beyond clinical applications, Koua and Kraak used the World Bank's Living Standards Measurement Survey to analyze factors indicating well-being and estimate health indicators. [20]

The cumulative nature of previous work has demonstrated the SOM as a tool to recognize patterns within data sets measuring clinical health outcomes, social and economic variables, and the physical environment. The algorithm's tolerance of nonlinear and nonparametric data presents an opportunity for the SOM methodology to recognize patterns among disease-causing variables within complex, multivariate data sets. Coupling the SOM algorithm's pattern recognition capabilities with the spatial analysis capabilities of geographic information systems (GIS) provides a novel approach to study how complex environmental influences affect health outcomes in populations.

The purpose of this study was to explore the potential of a coupled SOM-GIS approach to apply complexity theory to public health research, using community health assessment as an example. Such an approach would enable researchers to overcome challenges of non-linearity and skewed data distributions that have limited research efforts in the past. In this work we classified communities based on social, economic, and physical environmental factors using GIS and SOM methods. We present the results of our work and then discuss the challenges of implementing SOM-GIS for public health research.

## Results

### Self-Organizing Map Results

**Figure 1:**
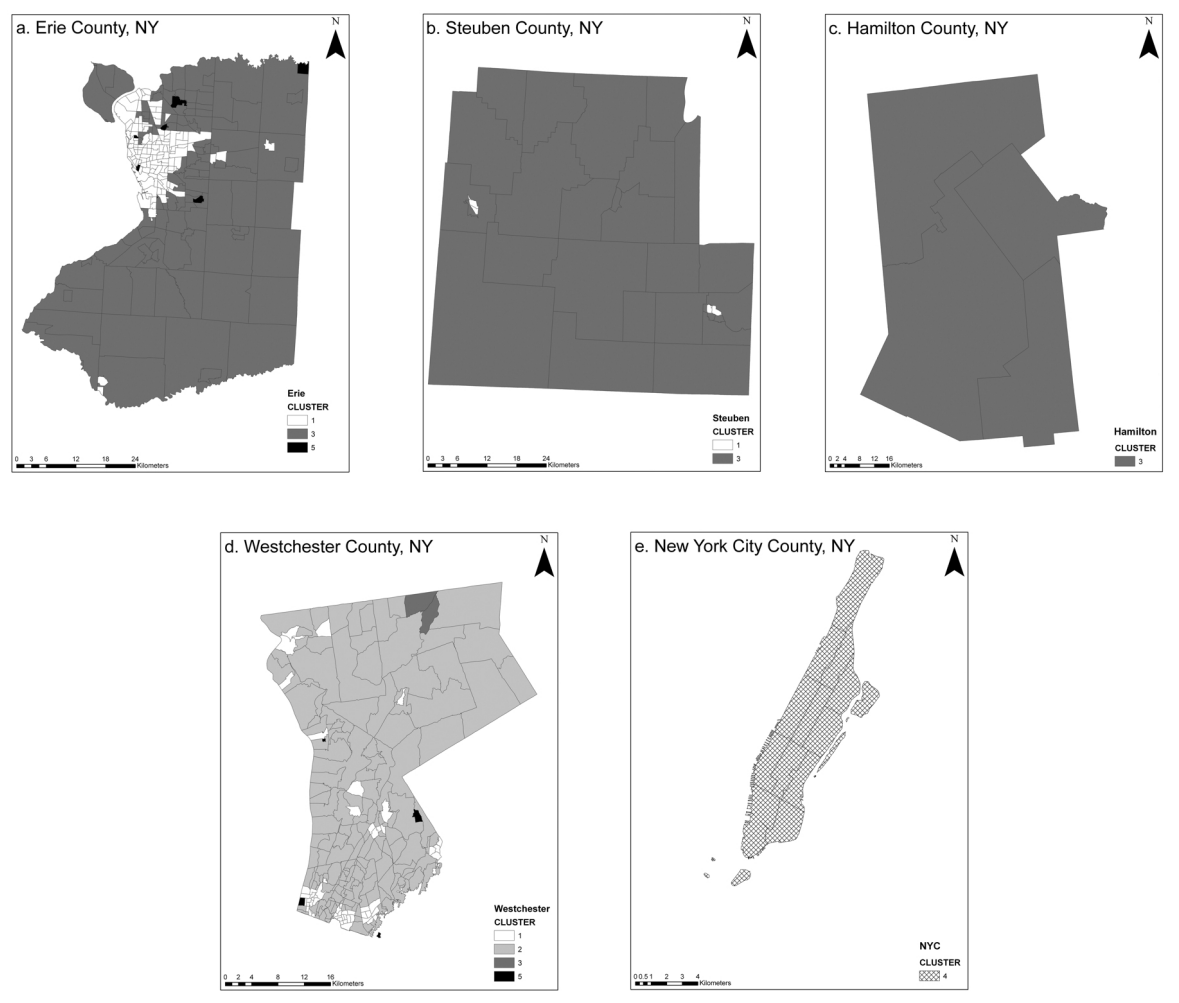
**SOM clusters in geographic space for five New York counties (A-E)**.

The SOM analyzed data describing ninety-two environmental variables for 511 communities representing five counties in New York State. Cluster tuning recognized five significant clusters and communities were categorized according to patterns discovered among variables. Figure 1 shows the geographic distribution of clusters by county. Cluster 1 included communities characterized by small to mid sized cities distributed throughout Erie, Westchester, and Steuben counties. Cluster 2 contained traditional suburban communities surrounding Buffalo, NY, and suburbs or small cities in Westchester County. Cluster 3 contained rural communities in Erie, Westchester, Steuben, and Hamilton counties. Cluster 4 included the highly urban communities of New York City County; and Cluster 5 represented a few communities in Erie and Westchester counties that were uninhabited or contained extremely few residents.

### Test for Spatial Autocorrelation

Moran's I was calculated in ArcGIS to test for spatial autocorrelation. Results provided Moran's Index equal to 0.24 and the associated p-value indicated a weakly significant result. The scale is from -1 to 1 where values near 1 are more clustered. Based on this test, the data indicated a low level of positive spatial correlation between the community clusters and, as such, values near one another were similar but not highly dependent on each other for their distribution. Spatial autocorrelation results suggest that major variables were most likely not omitted from the model, as a large Moran's Index indicates the potential for an incomplete model.

### Analysis of Variance

ANOVA evaluated the variance between the weighted observations of disease in communities and the cluster categories assigned to each of the communities (Table 1). Results indicated that significant differences exist between cluster classes and that there is more variation between clusters than the variation of disease counts within them, demonstrating the value of the grouping variable. For k-1 = 4 and N-k = 506 degrees freedom and Pr<0.01, the critical F value = 0.07. A significant result with a large ratio of between group variance to within group variance was observed for all health outcomes evaluated.

**Table 1:** Analysis of variance results according to health outcome.

		**DF**	**Sum Squares**	**Mean Square**	**F Value**	**Pr>F**	**R**^**2**^
**Outcome 1**	Treat	4	0.5953	0.1488	505.36	1	0.7998
Hepatitis A	Error	506	0.1490	0.0003			
**Outcome 2**	Treat	4	0.0138	0.0035	129.11	1	0.505097
TB	Error	506	0.0135	0.0000			
**Outcome 3**	Treat	4	6786.9301	1696.7330	193.14	1	0.604238
Asthma	Error	506	4445.2766	8.7851			
**Outcome 4**	Treat	4	692.6395	173.1599	326.14	1	0.720528
COPD	Error	506	268.6550	0.5309			
**Outcome 5**	Treat	4	0.0826	0.0206	111.02	1	0.4674
Fetal Immaturity	Error	506	0.0941	0.0002			
**Outcome 6**	Treat	4	3519.1946	879.7986	274.99	1	0.684921
Diabetes	Error	506	1618.9052	3.1994			
**Outcome 7**	Treat	4	1.9988	0.4997	171.27	1	0.575182
Influenza	Error	506	1.4763	0.0029			
**Outcome 8**	Treat	4	13301.8047	3325.4512	213.98	1	0.0628464
Atherosclerosis	Error	506	7863.7809	15.5411			
**Outcome 9**	Treat	4	33.4893	8.3723	2.67	0.97	0.020707
Alcohol & Drug	Error	506	1583.7708	3.1300			
**Outcome 10**	Treat	4	188.8172	47.2043	205.34	1	0.61879
Stress/Nonspec Dep	Error	506	116.3224	0.2299			

Lists the health outcomes studies with ANOVA results for k-1 = 4 and N-k = 506 degrees freedom and Pr<0.01, the critical F value = 0.07)

## Discussion

### Principal Findings

Our study demonstrated the potential of combining SOM and GIS to overcome traditional challenges associated with studying the complexities of the community environment. Kindig and Stoddart state that population health is fundamentally concerned with the interactions between multiple determinants of health outcomes, referring to such interactions as patterns. [21] Results indicated that the methods used were productive for determining the underlying mathematical patterns to group communities according to similar environmental characteristics. The integration of variables from multiple environmental components and the complex relationships considered to link such variables makes it difficult to uncover the significant relationships and sort out similar entities. By searching for patterns to group entities based on observed environmental conditions, it may be possible to discern characteristics of environments that influence community health status in future studies.

### Challenges Associated with Data Collection

Challenges for testing the hypothesis primarily surrounded obtaining data to represent environmental conditions and health outcomes. To satisfy requirements for the SOM input data needed to be either binary (0,1) or ratio level; additionally, geographic reference was necessary to connect variables with communities. Such conditions presented challenges for developing a diverse inventory of environmental variables since this study used secondary data from multiple sources. [22] The effect of pollutants on health is typically determined by exposure assessment, which is not an uncomplicated process. [23] For purposes of simplification the effects of pollutants were estimated with circular buffers. [24] Integration of exposure modelling within the SOM-GIS method is a natural next step that will improve the quality of input variables. [25] Another simplifying assumption was that Census variables from 2000 represented socioeconomic inputs for the five year period leading up to the Census survey. This assumption did not account for the dynamics associated with demographic variables such as migration or socioeconomic status. The necessity for health data also presented a substantial challenge. Hospital discharge data from the New York State Department of Health (NYSDOH) included conditions serious enough to require inpatient hospitalizations, but did not include data from outpatient services, minor emergency centres, or physician offices and clinics. The study design could not account for the latency between influence of environmental conditions and the onset of symptoms or disease and so the potential for patient migration between communities is of concern when using patient address to assign disease occurrences to communities.

### Unanswered Questions and Future Research

Our study presented methods that contribute to further research concerning the complexities of environmental systems and their relationship to human health outcome. [26,27,2] Using the SOM-GIS method, patterns relating a large number of variables and their interactions can be analyzed to group communities exhibiting similar data structures. If patterns are observed among the environmental conditions between communities and these patterns correspond significantly to the distribution of various diseases, several questions arise with numerous opportunities for future research. The overarching question is how can the mathematical patterns found among environmental variables be used to understand what is causing differences in observed rates of specific diseases? To determine how environmental conditions influence specific diseases, the variables (single or interactive group variables) that influence pattern structure should be identified. The context of these questions should also be explored to understand how the scale at which systems are studied modifies outcomes and also to determine the influence of nested hierarchical domains on observations at all scales. For example, what is observed at the individual level includes not only individual risk factors, but factors that operate at the population and regional scale, and the way these risk factors change through time. Within the need for contextual studies, research questions should consider the dynamical component of systems and include the temporal dimension to further understand latency between environmental effects and health outcomes.

## Conclusion

The significant relationship between SOM classifications and the geographic distribution of population-adjusted rates for selected diseases demonstrated a positive relationship between environmental conditions and health outcomes supporting previous work that described the environment as a determinant of population health. [28,29] This result provides observation-based credibility to conceptual theories suggesting that the environment functions as a complex system; and that environment is correlated with distributions of both chronic and infectious diseases in community level populations. [3,30,2] Given that environmental conditions are related to health outcomes, environmental variables may be useful in estimating population health. Multivariate environmental assessments may be used as proxies for practice-based health assessments in cases where data are limited. Further study is needed to determine the contribution of individual variables (or groups of variables), identify readily available data sets, and to fully investigate the development of a meaningful proxy measure.

## Methods

### Data Collection and Preparation

New York State has 62 counties that cover a wide range of landscapes, climate zones, industries, and socioeconomic populations . The counties were categorized based on the level of urbanization such that counties were grouped as highly urban, mixed urban, suburban, rural, and very rural to include a representative of each type of environment. One county was randomly selected from each of these groups; our analysis included five of the 62 counties (8%). The counties selected: New York City (Manhattan), Erie, Westchester, Steuben, and Hamilton.

To identify community boundaries U.S. Census tracts, considered homogeneous groups, were used for the upstate counties. Community boundaries within New York City were adapted from the New York City Department of City Planning. [31] Our intention was to select variables to represent the environmental factors described by previous conceptual models. [32] Environmental variables meeting the following requirements were collected from existing state-wide data sets for the 1995–2000-study period:

1. Ratio level observations,

2. Spatially referenced,

3. Consistent for all counties studied,

4. Measured at the community level.

**Figure 2:**
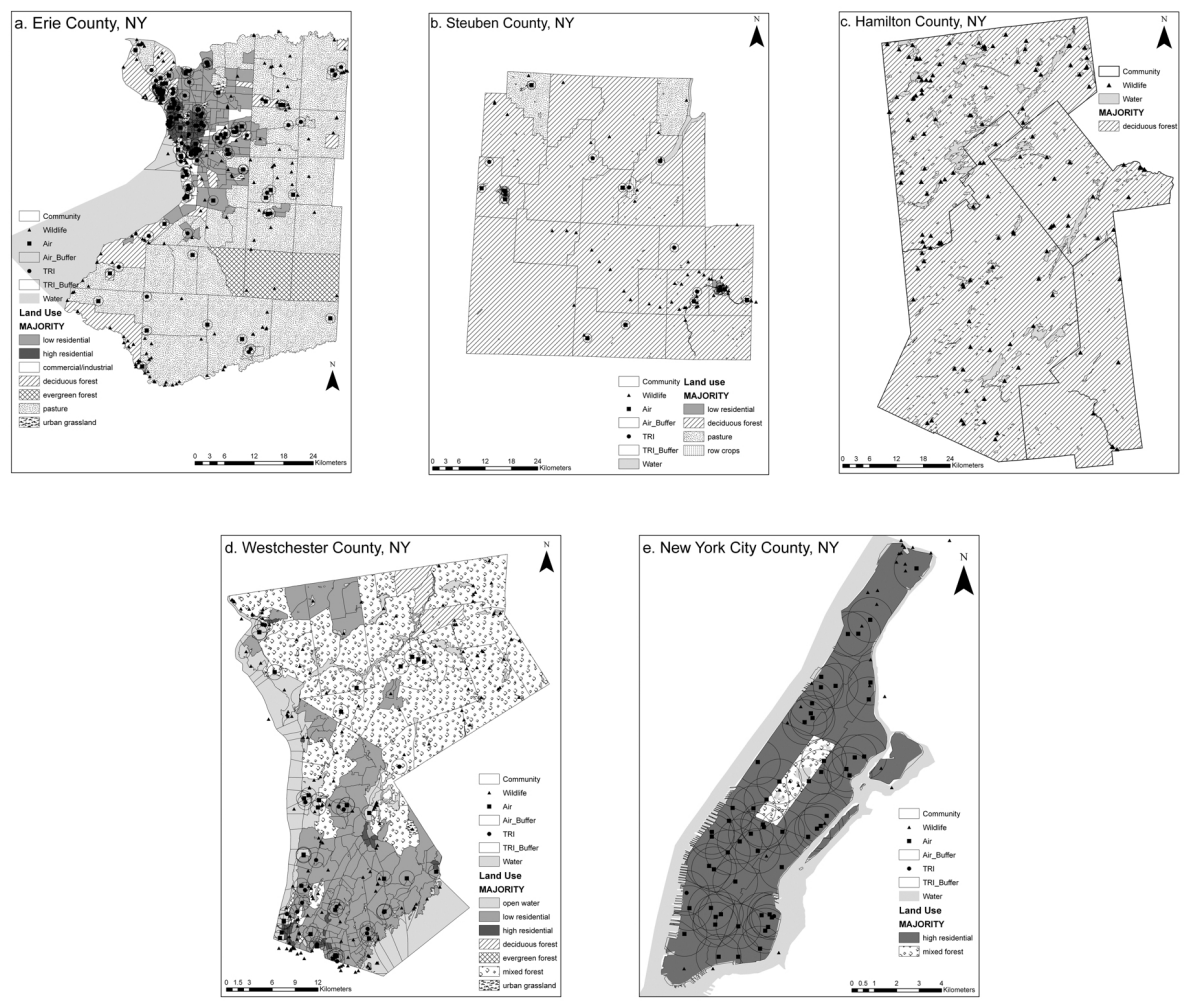
**Geographic distribution of input variables for five New York counties (A-E)**.

Data sets as input variables for the SOM included physical, economic, occupation, housing, education, and demographic information (Table 2). Figure 2 shows the geographic distribution of physical variables [including land use and Toxic Release Inventory (TRI)]. Data were formatted and pre-processed using SAS, Microsoft Excel, or ArcGIS to achieve spatial and temporal compatibility.

**Table 2:** Polymorphisms and substitution rates in the L, M and S sequences amplified from field-infected mosquitoes.

**Environment**	**Variable**	**Description**	**Source**
Physical	Air Quality	CO, NO, SO2, PM10, VOCU, PMU	NYSDEC Air Emission
Physical	Toxic Release	TRI Waste (Air & Water)	US EPA Toxic Release Inventory
Physical	Rare Species	Presence & Rank of Rare Species	NYSDEC Natural Heritage Inventory
Physical	Land Use	Majority Class (11 21 22 23 41 42 43 81 82 85)	USGS NLCD
Economic	Household Income	Income Distribution	US Census ESRI CommunityInfo
Economic	Rent Amount	Average Contract Rent	US Census ESRI CommunityInfo
Economic	Home Value	Average Value Owner Occupied Housing	US Census ESRI CommunityInfo
Occupation	Work Location	Within County of Residence	US Census ESRI CommunityInfo
Occupation	Transportation	Mode to Work (Auto, Carpool, Public, Bicycle, Walk)	US Census ESRI CommunityInfo
Occupation	Travel Time	Travel Time to Work	US Census ESRI CommunityInfo
Occupation	Industry	Industry of Employment (20 Classifications)	US Census ESRI CommunityInfo
Housing	Age of Housing	Year of Construction (Decadal Increments)	US Census ESRI CommunityInfo
Housing	Num Households	Total Households	US Census ESRI CommunityInfo
Housing	Household Size	Average Household Size	US Census ESRI CommunityInfo
Housing	Owner Occupancy	Owner vs. Renter Occupancy Ratios	US Census ESRI CommunityInfo
Housing	Vacant Housing	Vacant Housing Units	US Census ESRI CommunityInfo
Education	Education	Education Attainment (None thru Doctoral Degree)	US Census ESRI CommunityInfo
Demographic	Race	Racial Group (7 Categories)	US Census ESRI CommunityInfo
Demographic	Family	Family Status (Married, Children in Household), Size	US Census ESRI CommunityInfo
Demographic	Median Age	Median Age	US Census ESRI CommunityInfo
Demographic	Population Size	Total Population	US Census ESRI CommunityInfo

Toxic Release Inventory (TRI) maintained by the U.S. E.P.A. included self-reported releases and waste management activity for industrial facilities. Source locations were geocoded using longitude and latitude coordinates provided with the data and cross referenced with facility addresses to ensure positional accuracy. The reported discharge amounts were summed and added as an attribute for each facility in GIS.

Air pollution data was obtained from the New York State Department of Environmental Conservation (NYSDEC) for permitted stationary industrial facilities. Locations were geocoded using address and the accuracy was checked using Google Earth. Facilities ranged from large industrial sources such as refineries and chemical manufacturers to small businesses such as dry cleaning operations and filling stations. Data was originally collected to monitor for permit compliance and contained the amount of each pollutant discharged by location for each year for carbon monoxide, sulphur dioxide, and oxides of nitrogen, volatile organics, total particulate matter, and PM_10_. Annual emission amounts were summed for each of the pollutants by facility and added as an attribute to the facility location in GIS.

The impact area of chemical releases by both TRI and air sources were approximated with a 1-km^2 ^circular point buffer around the facility. [33,24] The spatial fraction of the buffer contained within the community was multiplied by the discharge quantity from the source. For example, if "Facility A" discharged 10,000 pounds of chemical, and .68 of the 1-km^2 ^area was contained within Community 1, an estimated 6800 pounds of chemical discharge was allocated to Community 1. Other communities containing the remaining fraction of Facility A's effect region were assigned the remaining fraction(s) of the emission using the same method. The circular buffers can be seen in Figure 2.

The status of rare species and land use represents the ecosystem related data. Land use data were obtained via remote sensing from the United States Geological Survey (USGS). Land use type and percent cover by type were calculated for each community using the zonal statistics function in ArcGIS. The majority land use type is shown in Figure 2, represented by shading of communities.

The presence and quality of rare species within a community was selected as a variable to approximate the level of biodiversity, an indicator of ecosystem health. [27] The NYSDEC, Natural Heritage Inventory (NHI) monitors 174 natural community types, 727 rare plant species, and 432 rare animal species across New York, keeping track of more than 11,900 locations where these species and communities are found . The database includes detailed information on the relative rareness of each species and community, the quality of their occurrences, and descriptions of sites. Data were provided in a de-identified format so that occurrences were listed by location and quality (see website for listing and definition of categories), but the scientific and common names of the organism were omitted for protection. Occurrences were ranked and weighted according to the quality reported by NHI, and the number of ranked occurrences per community was summed in ArcGIS. For example, the presence of a rare species was counted as 1, with added value for the ranked quality of the specimen.

ESRI's CommunityInfo product provided the 2000 U.S. Census SF3 survey data to represent the social, cultural, economic, educational, and occupational components of the communities. Each group of variables contained measures of subcategory variables (Table 2). Given that the study period was 1995–2000, the 2000 Census data were considered representative of the years preceding and leading up to the survey. Every community (such as a Census Tract) was assigned an identification number that was used to join variables using ArcGIS. The resulting table listed each community as a row with corresponding environmental variables occupying the columns.

### Self-Organizing Map Analysis

Data were imported from ArcGIS to Viscovery SOMine  for analysis. The number of input variables was multiplied by ten to establish the number of map nodes at 920; map tension influences the neighbourhood radius around nodes and was set at 0.2. [34] Analysis began with map training, or the gradual adaptation of nodes on the grid to resemble the underlying shape of the distribution. Thus, the order of nodes reflected the mathematical neighbourhood inherent in the data. Map training included searching for data clusters, retrieving numerical information, and calculation of cluster statistics. Cluster tuning used the significant breaks between groups of nodes to determine the number of clusters. Results were visualized as a two-dimensional hexagonal grid (or map) that indicated the relationship between nodes and displayed the distribution of data according to clusters. Each community was assigned to a data cluster based on the patterns observed for each corresponding variable. The geographic distribution of community clusters was mapped in ArcGIS using the community ID number to provide spatial reference.

### Health Data

The New York Data Protection Review Board reviewed and approved the use of data from the NYSDOH SPARCS inventory. This project was subject to additional review and approval by the University of Oklahoma IRB for human subjects research prior to the use of health information for this study. All data were used in a manner compliant with agreements between investigators and the NYSDOH and OU IRB.

Ten diseases were selected from the SPARCS database [35] to include infectious and chronic conditions (these are indicated on Table 1 in the results section). Disease occurrences were selected using ICD-9 codes, unique personal identifier codes, county, and year; and data were geocoded using patient address from the medical record. The observed frequency of each disease for every community was scaled according to community population and area density ratios. Analysis of variance (ANOVA) was conducted in SAS to test the relationship between disease frequency and community classification, assuming that all clusters had an equal opportunity for occurrence of disease.

## Competing interests

The authors declare that they have no competing interests.

## Authors' contributions

HB and MY collaborated on the study design, geographical analysis, and statistical analysis. HB obtained and preprocessed data, conducted SOM analysis and drafted the manuscript. MY also helped to draft and edit the manuscript.
